# Tumor Microenvironment-Triggered Dual-Prodrug Nanoplatform for Augmented Photodynamic Therapy: Self-Quenched Photosensitizer Activation and Nitric Oxide-Mediated Sensitization

**DOI:** 10.3390/polym18202606

**Published:** 2026-10-21

**Authors:** Yuxuan Gu, Jiaqi Ding, Jialin Zhang, Xiaoxuan Liu, Zhuotao Xu, Jimin Zhang, Xiongwei Qu

**Affiliations:** Hebei Key Laboratory of Functional Polymers, School of Chemical Engineering and Technology, Hebei University of Technology, Tianjin 300401, China

**Keywords:** dual-prodrug nano-assembly, photosensitizer activation, NO-mediated hypoxia alleviation, self-reinforcing ROS-cascade amplification, photodynamic therapy

## Abstract

Photodynamic therapy (PDT) holds great promise for cancer treatment owing to its spatiotemporal selectivity and minimal invasiveness. However, its therapeutic efficacy is hampered by photosensitizer self-quenching and intrinsic tumor hypoxia. Herein, we construct a tumor-microenvironment (TME)-activated dual-prodrug nanosystem (PheoA-CSSE-JSK) integrating the photosensitizer prodrug PheoA and nitric-oxide (NO) prodrug JSK for targeted tumor delivery. Tumor-abundant glutathione (GSH) cleaves disulfide bonds to restore the photoactivity of PheoA, which generates reactive oxygen species (ROS) under near-infrared irradiation. Meanwhile, intracellular GSH/glutathione S-transferase (GST) triggers NO release from JSK. NO-derived reactive nitrogen species (RNS) can degrade the tumor collagen matrix, which is inferred to potentially relieve hypoxia and further boost ROS production. GSH consumption, together with NO-mediated inhibition of the mitochondrial respiratory chain, may further augment ROS levels. We propose a self-reinforcing ROS-amplification working model involving PheoA photoactivation, potential NO-associated hypoxia alleviation, GSH depletion, and mitochondrial respiratory-chain inhibition. Collectively, these combined effects facilitate mitochondrial-dependent apoptosis and prominent antitumor outcomes. This TME-responsive dual-prodrug nanoplatform provides a promising strategy to augment PDT against hypoxic tumors.

## 1. Introduction

Photodynamic therapy (PDT) is gaining increasing interest in cancer treatment [1,2] due to its precise targeting and minimal invasiveness [3,4,5] as it can effectively destroy tumors while sparing healthy tissue [6,7]. PDT relies on three key components: a photosensitizer (PS), light, and oxygen [8,9,10]. Upon light irradiation, the PS converts oxygen into reactive oxygen species (ROS), which kill tumor cells by oxidizing cellular membranes [11,12,13]. However, the hydrophobic nature and poor tumor selectivity of many PS molecules compromise PDT efficacy and raise safety risks for healthy organs [14,15,16]. To address these limitations, delivery vehicles such as liposomes and micelles have been developed for PS delivery [17,18], yet they frequently suffer from reduced phototoxicity [19,20]. Therefore, there remains an urgent demand for nanosystems that maintain PS in a self-quenched state during systemic circulation.

Moreover, hypoxia represents another hallmark of most solid tumors [21,22,23], which greatly impedes the oxygen-dependent therapeutic efficiency of PDT [24,25]. Collagen, a major constituent of the tumor extracellular matrix [26,27], is a critical contributor to tumor hypoxia [28,29]. Accordingly, agents capable of degrading collagen to alleviate hypoxia are highly desirable for enhancing PDT outcomes. Nitric oxide (NO), an important gaseous signaling molecule, modulates tumor physiology and the tumor microenvironment [30,31,32]. Recent studies have demonstrated that NO can promote collagen degradation in tumors via reactive nitrogen species (RNS) [33,34]. Nevertheless, both NO and PS are small molecules prone to off-target side effects [35,36], making their site-specific release within tumor tissue challenging [37,38,39]. In this context, tumor-microenvironment (TME)-responsive nano-prodrug delivery systems constitute an innovative strategy to achieve tumor-confined NO and photosensitizer release and maximize their biological effects [40,41,42].

**Scheme 1:**
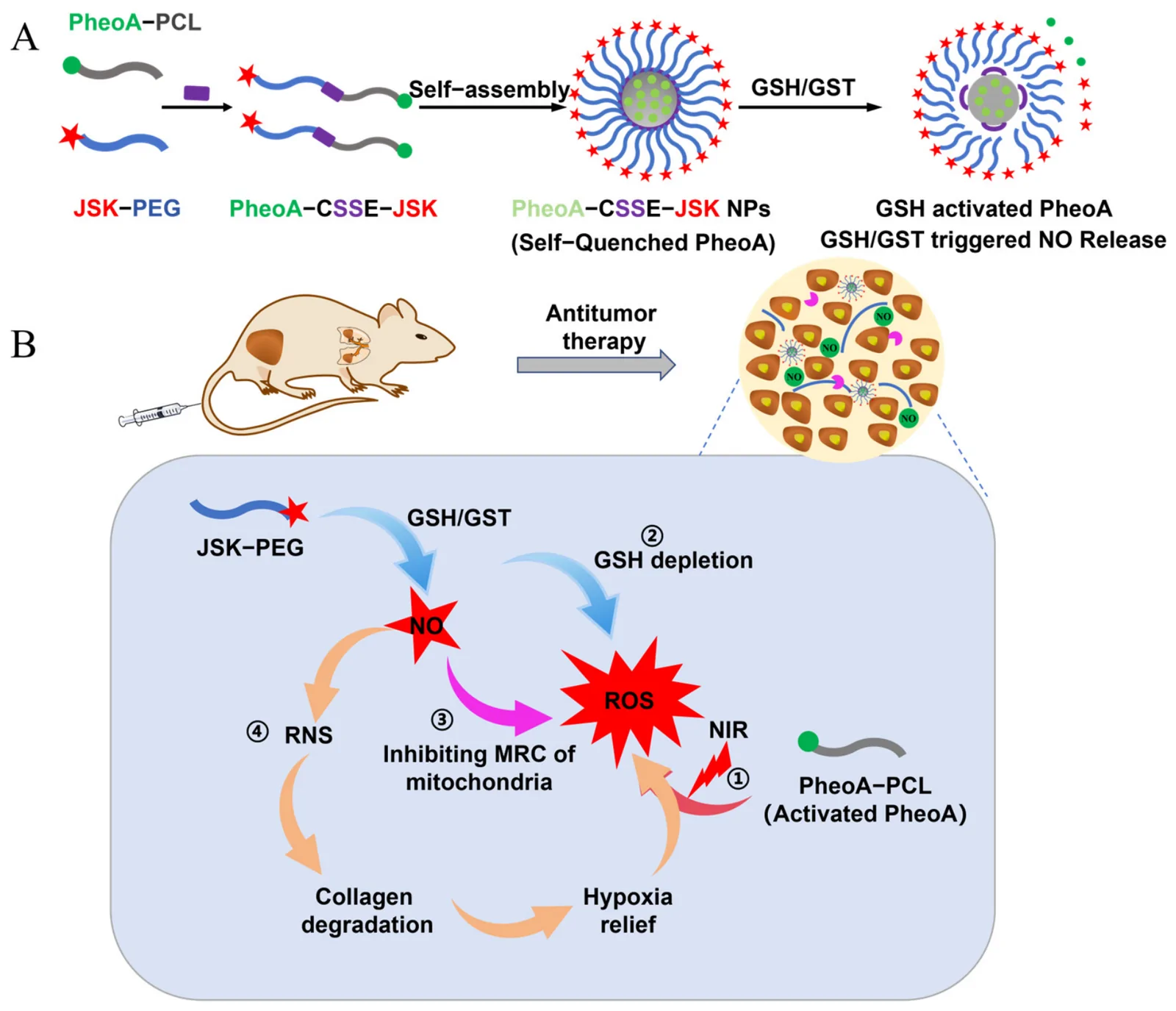
Schematic illustration of the TME-bioactivatable nano-prodrug for augmented antitumor efficiency. (**A**) Synthesis and self-assembly of the nano-prodrug PheoA-CSSE-JSK, featuring GSH-triggered PheoA activation and GSH/GST-stimulated NO release within the TME. Green spheres represent the photosensitizer prodrug PheoA, red pentagrams denote the JSK prodrug, gray lines represent the PCL polymer, blue lines represent the PEG polymer, and purple segments indicate the linker compound dialkynyl-Cys. (**B**) Mechanism of the TME-bioactivatable nano-prodrug for augmented photodynamic efficiency, including self-quenched photosensitizer activation, PheoA photoactivation, potential NO-associated hypoxia alleviation, GSH depletion, and mitochondrial respiratory-chain inhibition.

This study reports on a novel TME-triggered dual-prodrug nanosystem to improve PDT performance via self-quenched photosensitizer activation and NO-mediated therapeutic sensitization Scheme 1. The nanosystem, designated PheoA-CSSE-JSK, incorporates a self-quenched photosensitizer prodrug (PheoA) and a GSH/GST-responsive NO prodrug (JSK). PheoA and JSK are covalently tethered to the GSH-responsive copolymer CSSE, yielding nanoparticles with a hydrophilic shell and hydrophobic core that prevent nonspecific NO release. Inside tumor cells, elevated GSH concentrations cleave disulfide bonds to activate PheoA for ROS generation under NIR light. Simultaneously, overexpressed intracellular GSH/GST activates JSK to enable sustained NO release. NO-derived RNS is anticipated to degrade dense tumor collagen, thereby alleviating hypoxia and further promoting ROS production. Intracellular ROS levels are additionally elevated through GSH depletion and NO-mediated suppression of the mitochondrial respiratory chain. This multi-modal strategy for amplifying the ROS cascade is expected to counter major PDT limitations, including photosensitizer inactivation, hypoxia-driven therapeutic resistance, and ROS scavenging, ultimately yielding potent antitumor activity.

## 2. Materials and Methods

### 2.1. Materials

Pheophorbide A (PheoA); NH_2_-PEG-N_33_ (M_w_ = 2000); 1-(3-dimethylaminopropyl)-3-ethylcarbodiimide hydrochloride (EDC); N-hydroxysuccinimide (NHS); CuS_44_; sodium L-ascorbate; DCC; DMAP; cystamine dihydrochloride; 4-pentynoic acid; GSH; and GST were purchased from Beijing Solarbio Science & Technology, Beijing, China. 2-(4-amidino-phenyl)-6-indolecarbamidine dihydrochloride was obtained from Sigma-Aldrich, Inc., Saint Louis, MO, USA. Various assay kits and antibodies, including the Griess Reagent kit, NO fluorescence probe (DAF-FM DA), GSH & GSSG assay kit, Annexin V-FITC Apoptosis Detection Kit, Mitochondrial Membrane Potential assay kit, and ATP assay kit, as well as Rabbit Anti-3-Nitrotyrosine Antibody (bs-8551R), Rabbit Anti-HIF-1α Antibody (bs-0737R), Rabbit Anti-MMP-2 Antibody (bs-20705R), Goat Anti-Rabbit IgG H&L/Alexa Fluor 594 Antibody (bs-0295G-AF594), and normal goat serum (10%, C01-03001), were supplied by Beyotime Biotechnology, Shanghai, China. All animal experiments were performed strictly in accordance with the Guide for the Care and Use of Laboratory Animals (Ministry of Science and Technology of the People’s Republic of China, Policy No. 2006398) and were approved by the Animal Care and Use Center of Hebei University of Technology, Tianjin, China (approval No. HEBUTacvc2023048, approval date: 2023.6.26).

### 2.2. Synthesis and Characterization of PheoA-CSSE-JSK

JSK-PEG was first synthesized via click chemistry [43,44]. Briefly, N_3_-PEG-N_3_ (100 mg, 0.05 mmol) and alkynyl-JSK (20 mg, 0.04 mmol) were dissolved in tetrahydrofuran (THF, 6 mL) under nitrogen atmosphere. An aqueous solution containing CuSO_4_ (4 mg, 0.016 mmol) and sodium ascorbate (8 mg, 0.040 mmol) was added to the reaction mixture, which was incubated at 37 °C for 24 h. After reaction completion, crude JSK-PEG was purified by dialysis against deionized water. Subsequently, PheoA-PCL was synthesized. In brief, Pheophorbide A (PheoA, 30 mg, 0.05 mmol), NH_2_-C_2_H_4_-SS-C_2_H_4_-NH_2_ (7.6 mg, 0.05 mmol), N_3_-PCL-COOH (100 mg, 0.05 mmol), NHS (13 mg, 0.11 mmol), and EDC (44 mg, 0.23 mmol) were mixed in dimethyl sulfoxide (DMSO, 5 mL) and reacted at room temperature for 24 h. PheoA-PCL was recovered after dialysis and freeze-drying. Dialkynyl-Cys was prepared by reacting cystamine dihydrochloride (0.416 g, 2.70 mmol) with 4-pentynoic acid (0.582 g, 5.94 mmol) in anhydrous dichloromethane in the presence of DCC (1.33 g, 6.46 mmol) and DMAP (0.792 g, 6.48 mmol) under nitrogen atmosphere. The mixture was magnetically stirred for 48 h. The resulting white precipitate was removed by filtration, and dialkynyl-Cys was isolated via column chromatography.

PheoA-CSSE-JSK was obtained through a two-step click-chemistry procedure. First, PheoA-PCL (100 mg, 0.038 mmol) reacted with dialkynyl-Cys (14 mg, 0.044 mmol), using CuSO_4_ (4 mg, 0.016 mmol) and sodium ascorbate (8 mg, 0.040 mmol) as catalysts in THF/H_2_O mixed solvent (3:1, *v*/*v*) under nitrogen at 37 °C for 24 h. The intermediate PheoA-PCL-Cys was further reacted with JSK-PEG (100 mg, 0.041 mmol), CuSO_4_ (4 mg), and sodium ascorbate (8 mg) at 37 °C for another 24 h. The final product, PheoA-CSSE-JSK, was purified by dialysis and freeze-drying. JSK-PEG, PheoA-PCL, dialkynyl-Cys, and PheoA-CSSE-JSK were characterized using ^1^H-NMR, ATR-FTIR, and UV–Vis spectroscopy, which were performed using a Bruker Avance III spectrometer (Bruker, Karlsruhe, Germany), a Bruker VERTEX 70 spectrometer (Bruker, Karlsruhe, Germany), and a Shimadzu UV-2600 spectrophotometer (Shimadzu Corporation, Kyoto, Japan), respectively.Thermal properties and crystallinity were assessed by differential scanning calorimetry (DSC) and X-ray diffraction (XRD).

### 2.3. Critical Micelle Concentration (CMC)

The critical micelle concentration (CMC) of amphiphilic PheoA-CSSE-JSK was determined by fluorescence measurement using pyrene as a hydrophobic probe [45,46]. In short, pyrene stock solution in acetone (0.4 mL, 6 × 10^−6^ M) was transferred into volumetric flasks, and acetone was evaporated overnight. Polymer solutions with serial concentrations ranging from 10^−3^ to 10^−8^ g/mL were added to yield a final pyrene concentration of 6 × 10^−7^ M. Samples were sonicated for 30 min and equilibrated in the dark on a shaker for 24 h. Excitation spectra (300–380 nm; emission = 370 nm) were collected on a PerkinElmer LS50B fluorescence spectrophotometer (PerkinElmer, Waltham, MA, USA), with slit width set to 10 nm and scanning speed at 240 nm/min. The intensity ratio I_335_/I_330_ was plotted against the logarithm of polymer concentration.

### 2.4. Preparation of PheoA-CSSE-JSK Nanoparticles

PheoA-CSSE-JSK nanoparticles were fabricated via solvent evaporation. Briefly, 5.00 mg of PheoA-CSSE-JSK was dissolved in 200 μL THF and added dropwise into 5 mL deionized water under continuous stirring until complete THF evaporation. The dispersion was filtered through a 0.45 μm filter. Nanoparticle size and morphology were characterized by dynamic light scattering (DLS) and transmission electron microscopy (TEM, Thermo Fisher Scientific, Waltham, MA, USA). UV–Vis spectroscopy was applied to quantify JSK and PheoA contents and calculate drug-loading content (DLC).

### 2.5. Intracellular NO Release

Cumulative NO release was quantified using the Griess assay, which detects diazo chromophore formation upon reaction between NO-derived products and Griess reagent. 4T1 cells were treated with blank, PheoA-CSSE, or PheoA-CSSE-JSK and incubated at 37 °C for 72 h. Cell culture supernatants were collected and analyzed with a Griess assay kit; absorbance was read at 540 nm on a microplate reader, and cumulative NO levels were calculated against a standard curve. Intracellular NO production in 4T1-Luc cells was further visualized using the DAF-FM DA probe via confocal laser-scanning microscopy and flow cytometry. Briefly, 4T1 cells (1 × 10^5^ cells per well) were seeded in 35-mm confocal dishes and cultured for 24 h. Cells were loaded with DAF-FM DA probe for 20 min and washed three times with ice-cold PBS. Cells were then incubated with PheoA-CSSE NPs or PheoA-CSSE-JSK NPs for 2, 6, or 12 h. After incubation, nanoparticles were removed; cells were washed with cold PBS, fixed in 4% paraformaldehyde, and counter-stained with DAPI. Fluorescence signals were acquired by confocal microscopy (Carl Zeiss, Jena, Germany) and flow cytometry.

### 2.6. GSH Depletion and ROS Generation of PheoA-CSSE-JSK

GSH consumption by PheoA-CSSE-JSK NPs was first evaluated under simulated tumor-microenvironment conditions (5 mM GSH and 5 μg/mL GST in PBS). Different mass ratios (1:5, 1:2, 1:1, and 2:1) of PheoA-CSSE-JSK NPs relative to GSH were tested, and residual GSH was quantified at 24 h using a GSH assay kit. Intracellular GSH depletion was further measured with the GSH & GSSG assay kit. 4T1 cells were treated with blank medium, PheoA-CSSE NPs, or PheoA-CSSE-JSK NPs (PheoA equivalent = 64 μM). After 24 h incubation, culture medium was discarded; cells were washed three times with PBS and harvested by trypsinization. Intracellular GSH and GSSG concentrations were determined by reading absorbance at 410 nm according to the kit protocol.

Intracellular ROS generation in 4T1 cells was monitored using the DCFH-DA probe (Beyotime, Shanghai, China). Cells were pre-incubated with DCFH-DA for 20 min, followed by treatment with blank control, PheoA-CSSE NPs, or PheoA-CSSE-JSK NPs (PheoA = 64 μM) for another 24 h. Cells were collected after PBS washing, and ROS-related fluorescence was measured using a fluorescence spectrophotometer.

### 2.7. MRC Complex Activity of PheoA-CSSE-JSK

The influence of PheoA-CSSE-JSK on mitochondrial respiratory chain (MRC) complex activity was determined using commercial MRC complex activity assay kits (Genmed Scientifics). 4T1-Luc cells were treated with PBS, PheoA-CSSE, or PheoA-CSSE-JSK for 24 h. Mitochondria were isolated and resuspended in mito-cito buffer. Mitochondrial proteins were extracted by three freeze-thaw cycles (−70 °C ↔ 37 °C). Total protein concentration was quantified with a BCA kit. The enzymatic activities of MRC Complex I and Complex IV were assayed; activity values were normalized to total mitochondrial protein content for each sample.

### 2.8. Disassembly and Activation of PheoA in PheoA-CSSE-JSK

X-ray photoelectron spectroscopy (XPS, Thermo Fisher Scientific, MA, USA) and UV–Vis spectroscopy were used to characterize the chemical state of PheoA within PheoA-CSSE-JSK nanoparticles. To mimic the tumor intracellular environment, PheoA-CSSE-JSK NPs were incubated with 10 mM GSH to trigger nanoparticle disassembly. UV–Vis absorbance and particle size were recorded at predefined time intervals. DPBF (1,3-diphenylisobenzofuran) was employed as a probe to assess singlet-oxygen generation (SOG) after nanoparticle disassembly and PheoA photoactivation. Three experimental groups were set: PheoA-CSSE-JSK NPs; PheoA-CSSE-JSK NPs plus NIR irradiation (660 nm, 0.2 W/cm^2^, 5 min, total 60 J/cm^2^); and PheoA-CSSE-JSK NPs pre-incubated with 10 mM GSH followed by identical NIR irradiation. DPBF stock solution (1 μL, 5 mg/mL in DMSO) was added to each sample. Time-dependent decay of DPBF absorbance at 404 nm was tracked on a microplate reader.

### 2.9. Detection of RNS

Dihydrorhodamine-123 (DHR-123, Beyotime S0019) served as a fluorescent probe for peroxynitrite anion (ONOO^−^) detection. Upon reaction with ONOO^−^, non-fluorescent DHR-123 is converted into fluorescent rhodamine-123 (emission = 526 nm). 4T1 cells were treated with PheoA-CSSE, PheoA-CSSE/NIR, PheoA-CSSE-JSK, or PheoA-CSSE-JSK/NIR (NP concentration = 0.05 mM; NIR: 660 nm, 0.2 W/cm^2^ for 5 min). Cell suspensions were collected, and fluorescence intensity was measured by a fluorescence spectrophotometer.

### 2.10. Evaluation of NO-Mediated Hypoxia Alleviation for PDT Sensitization

A 4T1 tumor-bearing mouse model was established by subcutaneous injection of 1 × 10^6^ 4T1-Luc cells into the right flank of female BALB/c mice. Treatments started once tumor volume reached ~100 mm^3^. Mice received intravenous injection of PheoA-CSSE, PheoA-CSSE/NIR, or PheoA-CSSE-JSK, PheoA-CSSE-JSK/NIR (100 μL; PheoA-based concentration = 64 μM). Seven days post-injection, mice were euthanized, and tumor tissues were harvested and fixed in 4% formaldehyde for histological examination. Tumor-tissue-associated RNS and ROS were probed using 3-NT antibody and DCFH-DA, respectively. The expression levels of MMP-2, HIF-1α, and collagen were analyzed via immunofluorescence and immunohistochemical staining.

### 2.11. Mitochondrial Membrane Potential Assay

Mitochondrial membrane potential was evaluated using the JC-1 assay kit. 4T1-Luc cells were seeded in 6-well plates (1 × 10^5^ cells per well) and cultured for 12 h. Culture medium was replaced with fresh medium containing PheoA-CSSE, PheoA-CSSE/NIR, PheoA-CSSE-JSK, or PheoA-CSSE-JSK/NIR (NP concentration = 0.05 mM; NIR: 660 nm, 0.2 W/cm^2^, 5 min, 60 J/cm^2^). After 6 h of incubation, cells were washed three times with PBS and incubated with JC-1 working solution for 30 min at 37 °C. After additional PBS washes, fluorescence images were acquired under a fluorescence microscope. Caspase-3 activity was measured to evaluate nanoparticle-triggered apoptosis. 4T1-Luc cells were seeded in 24-well plates and cultured for 24 h before receiving different treatments (PheoA-CSSE, PheoA-CSSE/NIR, PheoA-CSSE-JSK, PheoA-CSSE-JSK/NIR; NP = 0.05 mM; NIR: 660 nm, 0.2 W/cm^2^, 5 min, 60 J/cm^2^). Following 6 h treatment, cellular caspase-3 enzymatic activity was quantified using a caspase-3 assay kit.

### 2.12. In Vitro Cytotoxicity and Apoptosis Assays of PheoA-CSSE-JSK

In vitro cytotoxicity toward 4T1-Luc cells was determined using the CCK-8 assay. 4T1-Luc cells were seeded in 96-well plates (5 × 10^3^ cells per well) and exposed to serial concentrations (2, 4, 8, 16, 32, 64, 128, and 256 μM) of PheoA-CSSE, PheoA-CSSE/NIR, PheoA-CSSE-JSK, or PheoA-CSSE-JSK/NIR for 48 h. NIR irradiation (660 nm, 0.2 W/cm^2^; total dose = 60 J/cm^2^; and 5 min duration) was applied for corresponding groups. Cell viability was assessed by CCK-8 measurement. The cytotoxicity toward normal 3T3 fibroblasts was evaluated following identical procedures. Apoptosis was quantified with the Annexin V-FITC/propidium iodide apoptosis detection kit. Briefly, 4T1-Luc cells (1 × 10^5^ per well in 6-well plates) were treated with PheoA-CSSE, PheoA-CSSE/NIR, PheoA-CSSE-JSK, or PheoA-CSSE-JSK/NIR (JSK-based NP = 64 μM; and NIR: 660 nm, 0.2 W/cm^2^, 5 min, 60 J/cm^2^) for 24 h. Cells were harvested, stained with Annexin V-FITC and PI in the dark for 15 min, and analyzed by flow cytometry. Live/dead cell staining was also performed; fluorescence images were captured with an inverted fluorescence microscope to visualize direct nanoparticle-mediated cytotoxicity.

### 2.13. In Vivo Biodistribution and Antitumor Therapeutic Performance

The in vivo biodistribution and antitumor efficacy of nanoparticles were assessed using 4T1-Luc tumor-bearing BALB/c mice (initial tumor volume ≈ 100 mm^3^). Mice were intravenously injected with free PheoA/NIR, PheoA-CSSE/NIR, or PheoA-CSSE-JSK/NIR. Major organs and tumor tissues were collected 24 h post-injection to record PheoA autofluorescence signals. For antitumor efficacy evaluation, tumor-bearing mice were randomly divided into four groups (n = 5 per group): PBS, PheoA-CSSE/NIR, PheoA-CSSE-JSK, and PheoA-CSSE-JSK/NIR. Nanoparticles were administrated intravenously every three days (JSK = 5 mg/kg and PheoA = 7.5 mg/kg). After each injection, tumor sites received NIR irradiation (660 nm, 0.2 W/cm^2^, 5 min, and total 60 J/cm^2^). Tumor growth was monitored via bioluminescence imaging of 4T1-Luc cells. Tumor volume and mouse body weight were recorded periodically; tumor volume was calculated as V = (L* W^2^)/2, where L = longest tumor diameter and W = shortest tumor diameter (mm). On day 21, all mice were euthanized. Tumor tissues were excised for weight measurement and histological assessment. Tumor tissues were fixed in 4% formaldehyde, processed into 5 μm-thick sections, and subjected to hematoxylin-eosin (H&E) staining.

### 2.14. Statistical Analysis

Two-tailed Student’s *t*-test was used for comparisons between two groups. For experiments involving three or more experimental groups, one-way analysis of variance (ANOVA) followed by Tukey’s post hoc test was applied for multiple-group comparisons. All data are presented as mean ± standard deviation (SD). n = 3 indicates three independent biological replicates. Statistical significance was defined as * *p* < 0.05, ** *p* < 0.01, and *** *p* < 0.001.

## 3. Results and Discussion

### 3.1. Synthesis and Characterization of PheoA-CSSE-JSK

**Figure 1:**
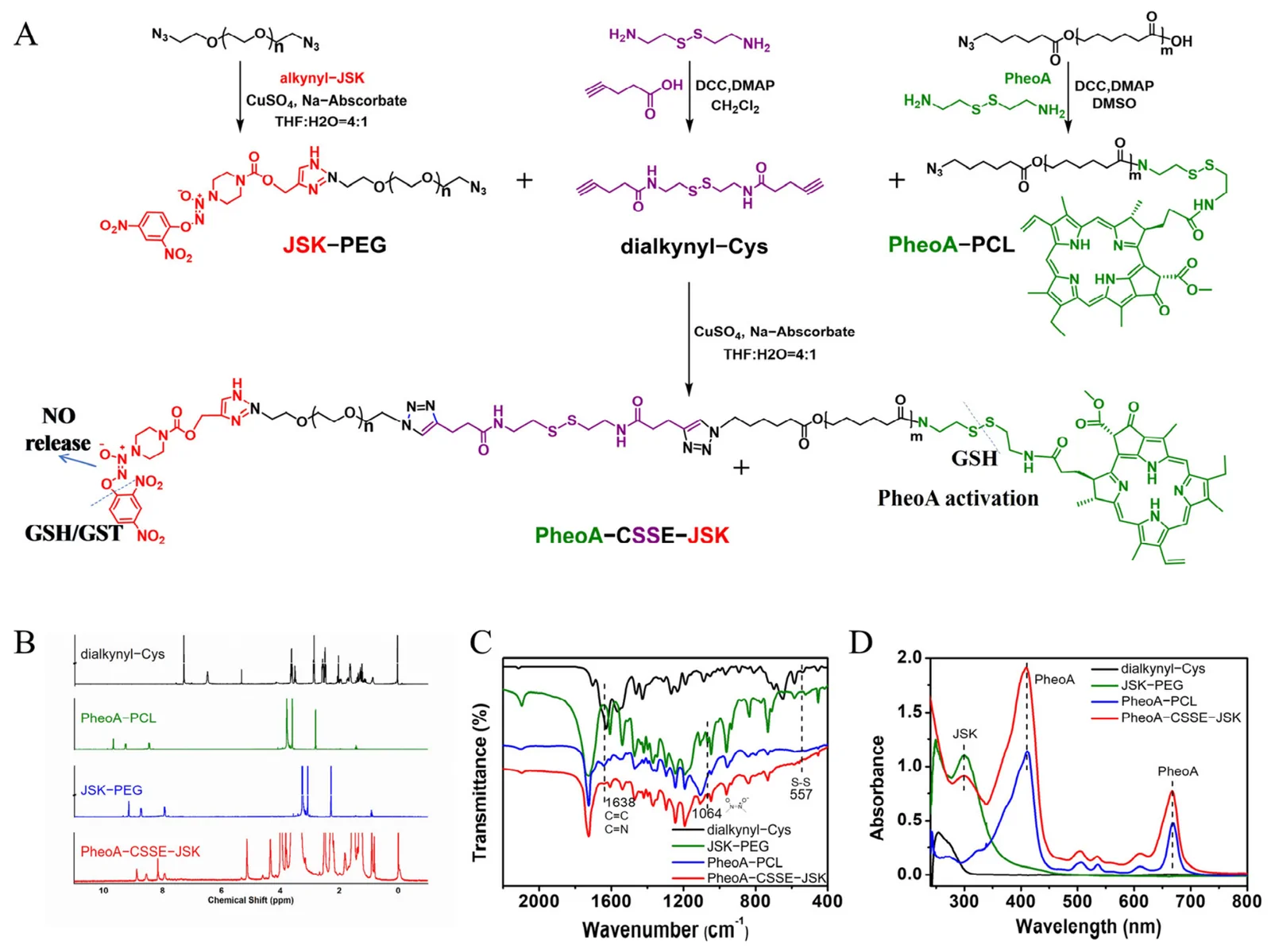
Synthesis and characterization of the photosensitizer-prodrug and NO-prodrug co-functionalized graft copolymer PheoA-CSSE-JSK. (**A**) Synthetic routes toward PheoA-CSSE-JSK. The polymer exerts two intracellular functions in tumor cells: GSH-triggered PheoA activation, where tumoral GSH cleaves disulfide bonds to restore PheoA photoactivity via NP disassembly and disrupted π–π stacking; and GSH/GST-triggered NO release from JSK, where tumoral GSH/GST cleaves JSK’s responsive linkage for sustained NO release. The red color represents the JSK prodrug, and the green color denotes the PheoA prodrug. (**B**) ^1^H-NMR, (**C**) ATR-FTIR, and (**D**) UV–Vis spectra of dialkynyl-Cys, JSK-PEG, PheoA-PCL, and PheoA-CSSE-JSK measured in DMSO.

The dual-prodrug graft copolymer PheoA-CSSE-JSK, functionalized with both NO prodrug JSK and photosensitizer prodrug PheoA, was designed and synthesized as illustrated in Figure 1A. The building blocks JSK-PEG, PheoA-PCL, and linker dialkynyl-Cys were individually synthesized and fully characterized. JSK-PEG was prepared by conjugating alkynyl-JSK onto N_3_-PEG-N_3_ via copper-catalyzed azide-alkyne cycloaddition click chemistry. ^1^H NMR spectra confirmed successful grafting of JSK onto PEG termini; the grafting efficiency was calculated as 52.31% by integrating aromatic protons of JSK (δ = 7.9–9.2 ppm) against characteristic PEG signals at δ = 3.25 ppm (-CH_2_-CH_2_-) (Figure S1). ATR-FTIR further supported JSK conjugation, showing partial attenuation of the azide stretching band near 2100 cm^−1^ (Figure S2). PheoA-PCL was obtained via amide coupling between PheoA and N_3_-PCL-COOH. Characteristic PheoA-derived resonances at δ = 8.5–9.7 ppm and PCL signals near δ = 3.7 ppm in ^1^H-NMR spectra allowed calculation of a PheoA grafting ratio of 90.22% (Figures S3 and S4). The GSH-responsive cross-linker dialkynyl-Cys was also synthesized and structurally validated by ^1^H-NMR and ATR-FTIR (Figures S5 and S6).

Subsequently, PheoA-CSSE-JSK was assembled by conjugating JSK-PEG and PheoA-PCL to the disulfide-containing dialkynyl-Cys linker through click-chemistry reactions, which are widely adopted for biomaterial preparation owing to high reaction efficiency and low toxicity [47]. ^1^H-NMR spectra of PheoA-CSSE-JSK exhibited diagnostic peaks assigned to PheoA (δ = 8–10 ppm) and the aromatic protons of JSK (δ = 7–8 ppm; Figure 1B). ATR-FTIR spectra (Figure 1C) displayed the diazeniumdiolate-related band of JSK at 1064 cm^−1^, C=C/C=N stretching vibrations of PheoA near 1638 cm^−1^, and S-S bond signals at 589 cm^−1^ and 557 cm^−1^. UV–Vis absorption spectra (Figure 1D) showed characteristic absorbance of JSK diazeniumdiolate (300 nm) and PheoA (670 nm), collectively verifying successful synthesis of PheoA-CSSE-JSK.

### 3.2. Thermodynamic Properties, Self-Assembly, and Nanoparticle Characteristics

**Figure 2:**
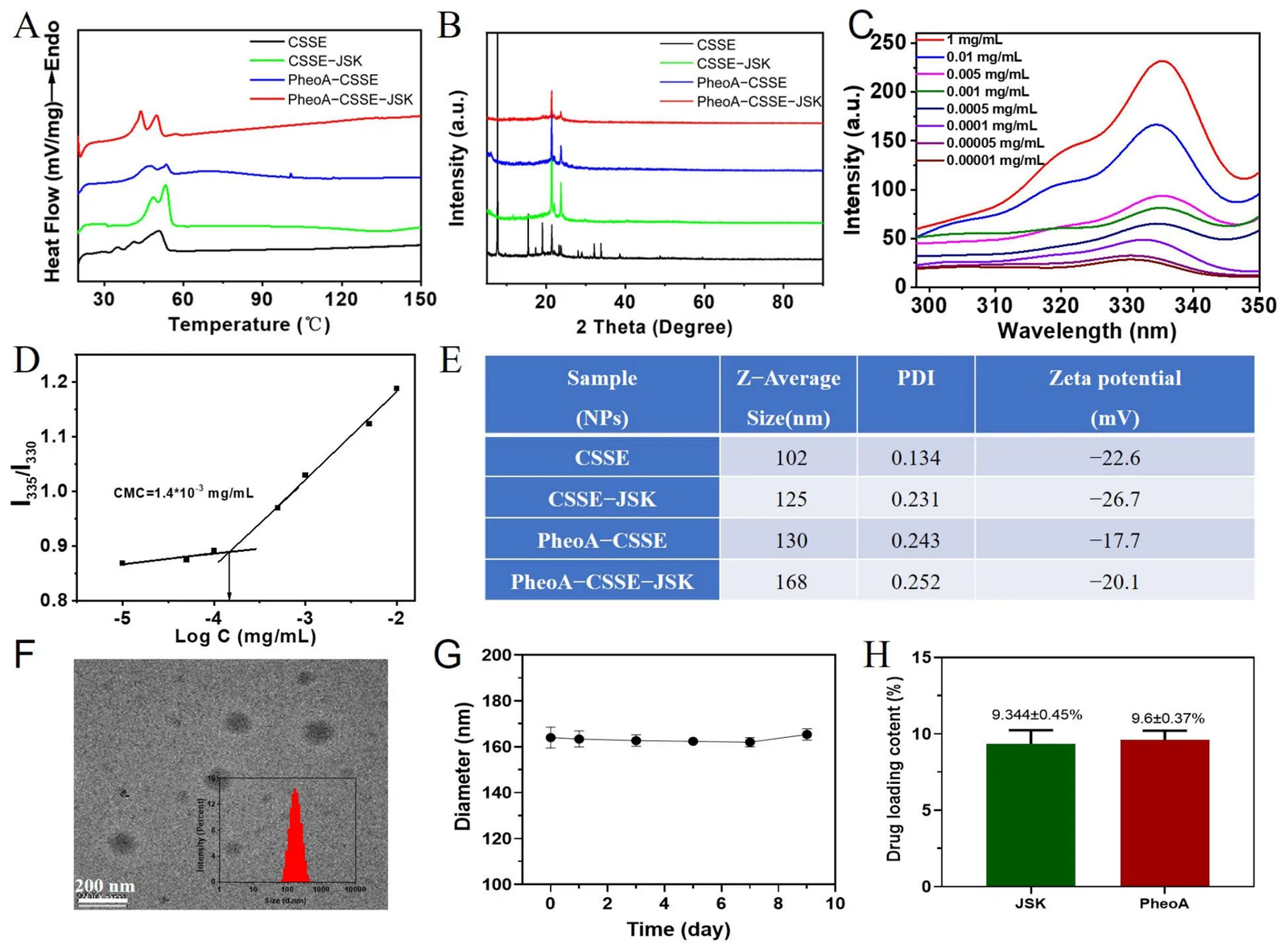
Thermodynamic profiles, self-assembly behavior, and nanoparticle physicochemical properties of PheoA-CSSE-JSK. (**A**) DSC thermograms and (**B**) XRD patterns of CSSE, CSSE-JSK, PheoA-CSSE, and PheoA-CSSE-JSK. (**C**) Pyrene excitation fluorescence spectra at different PheoA-CSSE-JSK concentrations. (**D**) CMC determination based on the fluorescence-intensity ratio I_335_/I_330_. (**E**) Summary table listing DLS-derived particle size, PDI, and zeta potential for CSSE NPs, CSSE-JSK NPs, PheoA-CSSE NPs and -PheoA-CSSE-JSK NPs. (**F**) TEM image and DLS size distribution of PheoA-CSSE-JSK NPs. (**G**) Time-dependent particle-size change in PheoA-CSSE-JSK NPs (1 mg/mL) incubated in PBS supplemented with 10% serum over 9 days. (**H**) Drug-loading content of JSK and PheoA within PheoA-CSSE-JSK NPs.

Thermal behavior and crystallinity of PheoA-CSSE-JSK were investigated by DSC and XRD. DSC heating curves revealed two melting transitions between 35 and 55 °C for all polymer samples, corresponding to the melting and recrystallization of PCL segments (Figure 2A). No obvious PEG-related melting endotherm was observed for PheoA-CSSE-JSK, presumably because the PCL-rich domain constrains polymer-chain mobility and suppresses PEG crystallization. The melting points T_m_ of PheoA-CSSE-JSK were recorded at 48.7 °C and 40.3 °C, which is lower than those of CSSE (50.2 °C and 53.4 °C), CSSE-JSK (40.3 °C and 47.3 °C), and PheoA-CSSE (49.4 °C and 53.4 °C). This reduction indicates decreased crystallinity in PheoA-CSSE-JSK, likely originating from the steric hindrance introduced by grafted JSK and PheoA moieties, which disturbs polymer-chain packing. Consistently, XRD patterns (Figure 2B) showed markedly diminished PCL-related diffraction peaks (2θ = 21.3°, 23.7°) for PheoA-CSSE-JSK relative to CSSE-JSK and PheoA-CSSE. Reduced crystallinity favors the formation of hydrophobic micellar cores during aqueous self-assembly. Critical micelle concentration (CMC) is a key metric evaluating amphiphilic-polymer self-assembly [48,49]. Herein, pyrene-probed fluorescence spectroscopy was used to assess the aggregation behavior of PheoA-CSSE-JSK. As polymer concentration increased over three orders of magnitude, pyrene fluorescence intensity rose, accompanied by a spectral red-shift from 330 nm to 335 nm (Figure 2C), indicating pyrene partitioning into hydrophobic micellar interiors. Plotting the intensity ratio I335/I330 against log(concentration) yielded a CMC value as low as 1.4 × 10^−3^ mg/mL for PheoA-CSSE-JSK NPs (Figure 2D). This value is considerably lower than that reported for bare PCL-PheoA nanoparticles (0.02 mg/mL), implying superior colloidal stability upon in vivo dilution.

Nanoparticles were prepared by nanoprecipitation. DLS-measured hydrodynamic diameters of CSSE, CSSE-JSK, PheoA-CSSE, and PheoA-CSSE-JSK were 102 nm, 125 nm, 130 nm, and 168 nm, with PDI values of 0.134, 0.231, 0.243, and 0.252, respectively (Figure 2E). Zeta potentials were −22.6 mV, −26.7 mV, −17.7 mV, and −20.1 mV; negative surface charges confer good colloidal dispersion. These self-assembled nanoparticles possess favorable particle dimensions and relatively narrow size distributions, which are desirable traits for drug-delivery applications. TEM micrographs demonstrated spherical morphology for PheoA-CSSE-JSK NPs with a dry-state diameter of ~134 nm (Figure 2F). The discrepancy relative to DLS (168 nm) arises from particle shrinkage upon dehydration during TEM sample preparation. The nanoparticles maintained stable hydrodynamic size over 9 days in PBS containing 10% serum (Figure 2G), demonstrating satisfactory serum-resistant colloidal stability. UV–Vis quantification gave drug-loading contents of 9.34% for JSK and 9.60% for PheoA within PheoA-CSSE-JSK NPs (Figure 2H).

### 3.3. Disassembly and Activation of Self-Quenched PheoA

**Figure 3:**
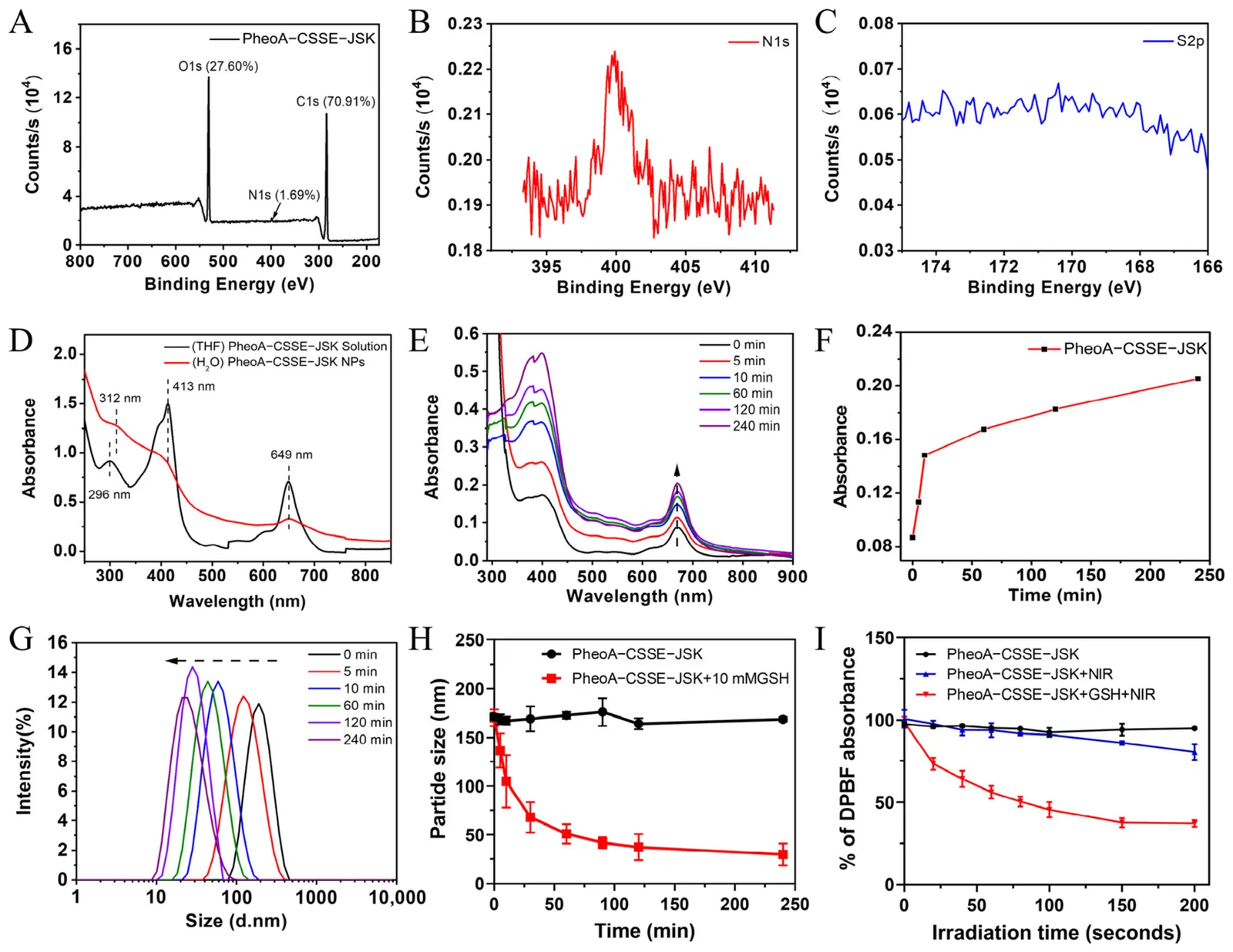
Disassembly and photoactivation of self-quenched photosensitizer. (**A**) Survey XPS spectrum of PheoA-CSSE-JSK nanoparticles; high-resolution (**B**) N 1s and (**C**) S 2p XPS spectra. (**D**) UV–Vis spectra of PheoA-CSSE-JSK in THF (molecularly dissolved) versus aqueous nanoparticle dispersion. (**E**) Time-evolution UV–Vis spectra of PheoA-CSSE-JSK NPs upon incubation with 10 mM GSH, the arrow indicates that more PheoA is disassembled as time extends. (**F**) Corresponding absorbance changes in PheoA, the arrow indicates that the nanoparticles gradually disassemble over time. (**G**,**H**) Time-dependent particle-size distribution and average hydrodynamic diameter of GSH-treated nanoparticles. (**I**) Relative residual DPBF absorbance as a function of NIR-irradiation time under different experimental conditions.

Physical encapsulation of NO donors inside nanoparticles frequently causes premature drug leakage and elevated off-target toxicity [50]. In our system, JSK is covalently grafted onto the hydrophilic PEG segment via a GSH/GST-responsive linkage to improve loading stability. XPS elemental analysis was performed to investigate the surface chemical composition of PheoA-CSSE-JSK NPs (Figure 3A–C). The N 1s signal at 399.71 eV displayed an atomic percentage of 1.34%, close to the theoretical value (1.2%) expected for surface-localized JSK moieties. By contrast, characteristic sulfur signals originating from the dialkynyl-Cys disulfide linker were not detected. Given the limited XPS sampling depth (~10 nm), this observation suggests that long hydrophilic JSK-PEG chains might shield the disulfide-containing linker toward the nanoparticle interior. The self-quenched status of PheoA was further validated by comparing the UV–Vis spectra acquired from THF (molecularly dissolved state) versus those acquired from the aqueous dispersion (nanoparticle state; Figure 3D). Upon transferring PheoA-CSSE-JSK from THF into water, absorbance at 649 nm decreased markedly, consistent with π–π stacking-driven aggregation and the self-quenching of PheoA within nanoparticle cores. This aggregated, quenched state is beneficial for reducing background phototoxicity during systemic circulation.

The incubation of PheoA-CSSE-JSK NPs with 10 mM GSH triggered the gradual restoration of PheoA absorbance over 240 min (Figure 3E,F), which was accompanied by dramatic particle-size reduction from 168 nm down to ~10 nm, indicating nanoparticle disassembly (Figure 3G,H). The singlet-oxygen probe DPBF was applied to assess photoactivity recovery. Under NIR irradiation alone, the PheoA-CSSE-JSK group exhibited minimal DPBF decay due to persistent PheoA self-quenching. After GSH-triggered disassembly, substantial DPBF absorbance loss was observed, confirming restored photosensitizing activity and enhanced singlet-oxygen generation (Figure 3I). Collectively, the GSH-responsive activation mechanism can be summarized as follows: photosensitizer prodrug PheoA is conjugated to the polymer backbone through a cystamine-derived disulfide linker. Elevated intratumoral GSH (2–10 mM, far higher than in normal tissues) acts as a nucleophile to cleave disulfide bonds, disrupting the hydrophobic core of PheoA-CSSE-JSK nanoparticles. This breaks the intermolecular π–π stacking responsible for PheoA self-quenching, induces nanoparticle disassembly, restores PheoA photoactivity, and enables efficient singlet-oxygen production under NIR light irradiation.

### 3.4. Intracellular NO Release

**Figure 4:**
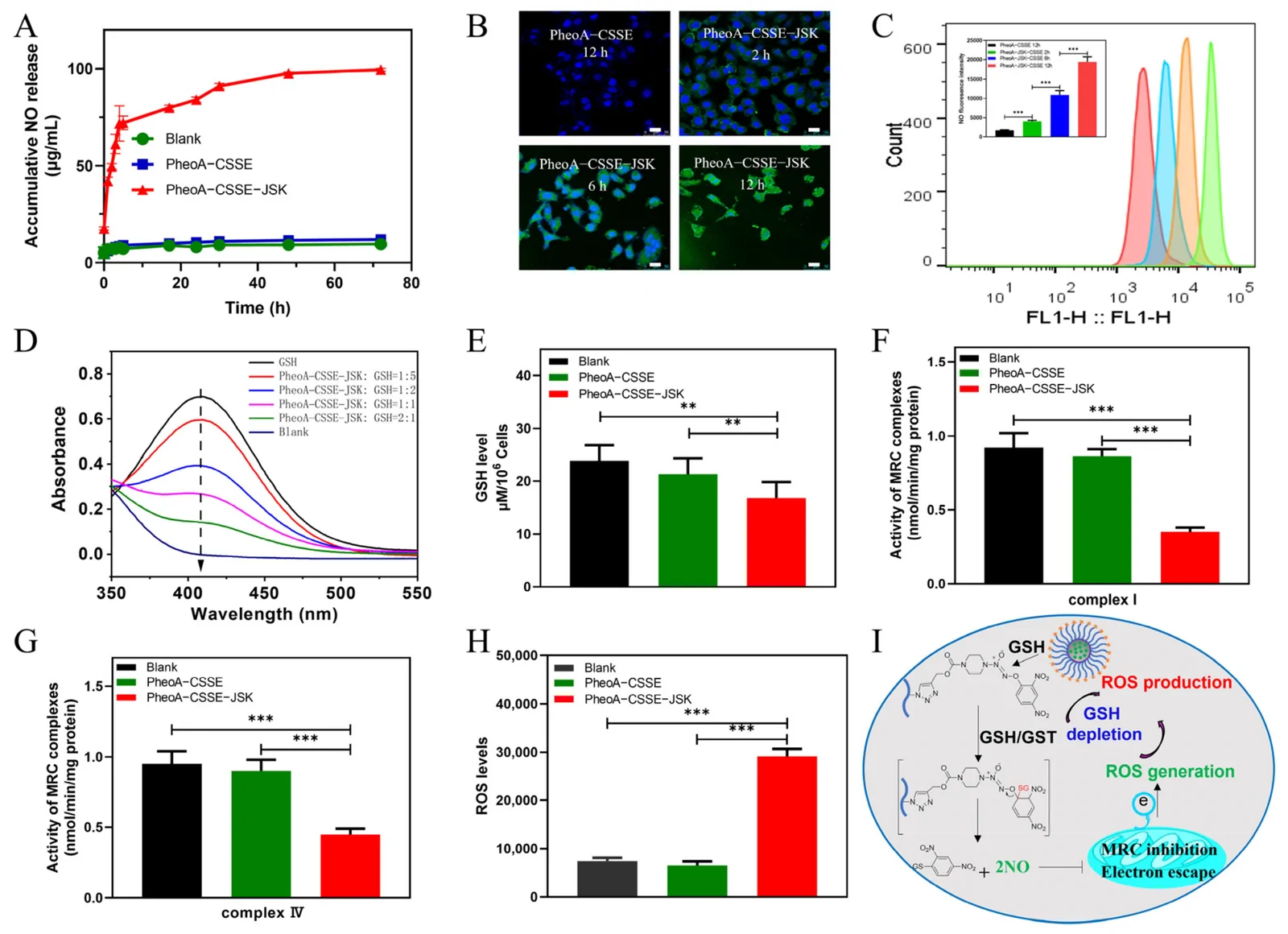
Intracellular NO release, GSH depletion and NO-mediated inhibition of mitochondrial-respiration-driven ROS elevation. (**A**) Cumulative NO release from different nanoparticle-treated 4T1 cells. (**B**) Confocal laser scanning microscopy and (**C**) flow cytometer fluorescence quantification of intracellular NO at 2 h, 6 h, and 12 h (NO, green and DAPI, blue), the color transition from red to green represents the gradual increase in NO fluorescence intensity; the scale length of each image is 25 μm. (**D**) UV spectrum for GSH detection in PBS with fixed 5 mM GSH and 5 μg/mL GST after treatment with different doses of PheoA-CSSE-JSK NPs, the arrow indicates that the GSH content gradually decreases with the increasing concentration of nanoparticles. (**E**) Intracellular GSH levels in 4T1 cells after 24 h nanoparticle treatment. (**F**) Activity of MRC complexes I and (**G**) activity of MRC complexes IV in mitochondria after nanoparticles treatment. (**H**) ROS levels after various nanoparticles treatment. (**I**) Schematic illustrating GSH-depletion and MRC-inhibition-driven ROS-amplification mechanisms. The data are expressed as mean ± SDs (n = 3). ** *p* < 0.01, and *** *p* < 0.001.

The NO prodrug JSK is tethered via a GSH/GST-susceptible carbamate-like linkage. Intracellular GSH (10 mM, which is considerably higher than in normal tissues) serves as a nucleophile, while tumor-overexpressed GST (5 μg/mL) catalyzes JSK decomposition to release two equivalents of NO. NO-derived bioactive species exert pronounced antitumor effects in multiple tumor models [51]. We, therefore, measured intracellular NO release using 4T1 tumor cells, which possess markedly higher GSH and GST levels relative to non-malignant cells. As shown in Figure 4A, PheoA-CSSE-JSK NPs that were mediated sustained NO accumulation in 4T1 cells, reaching 99.5 μg/mL at 72 h, and clearly outperforming the PheoA-CSSE and blank control groups. The DAF-FM-DA fluorescence probe was used for intracellular NO visualization. Weak cytoplasmic green fluorescence was detectable after 2 h incubation with PheoA-CSSE-JSK NPs. Fluorescence intensity increased at 6 h, with signals observed in both the cytoplasm and nucleus. At 12 h, maximum fluorescence intensity was accompanied by obvious cellular morphological shrinkage (Figure 4B). Flow-cytometry quantification further demonstrated progressive enhancement of DAF-FM-DA fluorescence intensity over time (signal counts: 3981 at 2 h; 12,100 at 6 h; and 20,502 at 12 h; Figure 4C). Such spatially confined, sustained NO release is favorable for improving therapeutic efficacy while preserving biosafety.

### 3.5. GSH Depletion and NO-Mediated Mitochondrial-Respiration Inhibition for Boosted ROS Generation

GSH functions both as a disulfide-cleaving agent for nanoparticle activation and a nucleophile initiating JSK-derived NO liberation [52]; consequently, GSH is consumed upon nanoparticle treatment. We first evaluated GSH-depletion capacity under cell-free simulated-tumor-microenvironment conditions. As the mass ratio of PheoA-CSSE-JSK NPs to GSH increased from 1:5 to 2:1, absorbance at 420 nm decreased, demonstrating effective GSH consumption (Figure 4D). Inside 4T1 cells, PheoA-CSSE-JSK treatment significantly lowered intracellular GSH levels compared with blank and PheoA-CSSE groups (Figure 4E), and the cellular GSH/GSSG ratio also declined (Figure S7). GSH depletion originates from two parallel processes: disulfide-bond cleavage and nucleophilic-substitution-driven NO release from JSK.

Mitochondria constitute the major cellular source of ROS, where the mitochondrial respiratory-chain (MRC) supplies electrons for oxygen-centered ROS formation [53]. To probe downstream ROS amplification triggered by PheoA-CSSE-JSK, we assessed MRC complex enzymatic activities. Among respiratory-chain complexes, Complex I (NADH-ubiquinone oxidoreductase), the principal mitochondrial superoxide source, can be persistently inhibited by NO-derived RNS via cysteine S-nitrosylation and iron-sulfur-cluster damage. Complex IV (cytochrome c oxidase), the terminal oxygen-consuming enzyme, undergoes reversible competitive inhibition by NO. Complex II and Complex III exhibit relatively low direct NO susceptibility. Accordingly, Complex I and Complex IV were selected to characterize NO-provoked respiratory-chain suppression. Relative to the PheoA-CSSE group, PheoA-CSSE-JSK treatment significantly suppressed Complex I and Complex IV activity (Figure 4F,G). As a combined outcome of GSH depletion and NO-mediated MRC inhibition, intracellular ROS abundance increased ~3.45-fold in the PheoA-CSSE-JSK group compared with the PheoA-CSSE-only group (Figure 4H). A schematic illustration summarizing ROS-amplification cascades is presented in Figure 4I.

### 3.6. NO-Mediated Hypoxia Alleviation for PDT Sensitization

**Figure 5:**
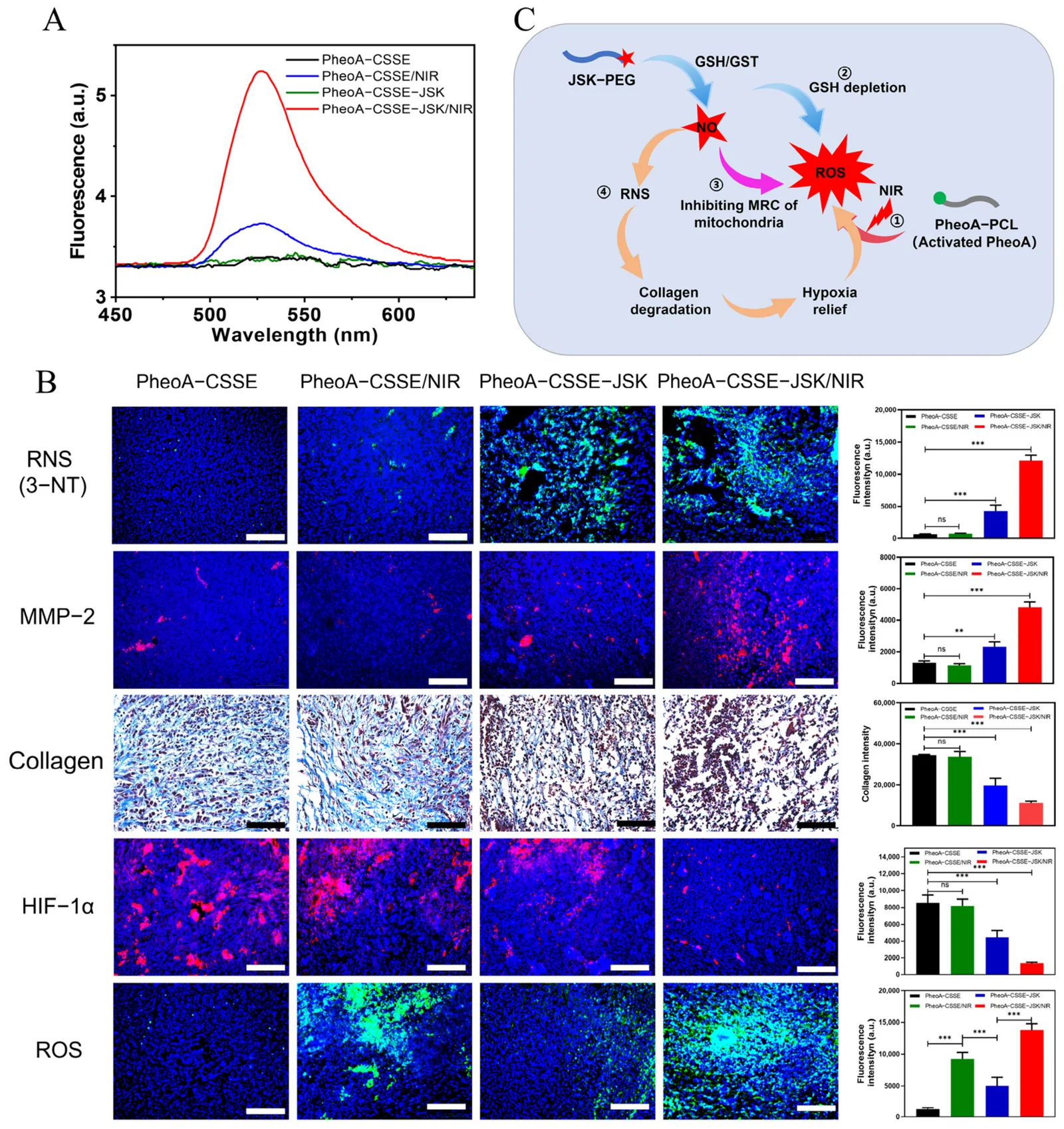
NO-mediated hypoxia alleviation for PDT sensitization. (**A**) In vitro cellular RNS generation monitored by DHR-123 fluorescence assay. (**B**) Representative immunofluorescence and histochemical staining of tumor sections for RNS (3-NT), MMP-2, collagen (Masson), HIF-1α, and ROS after seven-day in vivo treatments, together with corresponding quantitative fluorescence analysis. (**C**) Schematic diagram illustrating the positive-feedback cascade between RNS and ROS for NO-driven hypoxia relief. Data are given as mean ± SD (n = 3). ** *p* < 0.01, and *** *p* < 0.001. scale bar = 100 μm.

Abundant collagen within the tumor extracellular matrix hinders oxygen diffusion and establishes hypoxia, a well-recognized barrier limiting PDT therapeutic outcomes [54]. Recent work suggests that NO-derived RNS can promote tumor ECM collagen degradation, which may mitigate hypoxia and potentiate PDT. We first measured cellular RNS production in 4T1 cells. Negligible RNS-related fluorescence was observed for PheoA-CSSE and PheoA-CSSE/NIR groups, whereas obvious RNS signals appeared for PheoA-CSSE-JSK samples; RNS levels were further elevated upon NIR irradiation, reflecting the chemical reaction between NO and PDT-generated ROS (Figure 5A). These observations support a plausible mechanistic link between RNS-driven collagen breakdown and tumor-hypoxia alleviation, consistent with the previously published literature [55]. We infer that collagen degradation potentially improves intratumoral oxygen availability, which, in turn, contributes to lowered HIF-1α expression in tumor tissue.

In vivo tumor-tissue analysis was performed on 4T1 tumor-bearing mice seven days after different treatments (Figure 5B). RNS abundance was probed via 3-nitrotyrosine immunofluorescence. The PheoA-CSSE-JSK/NIR group displayed the strongest 3-NT fluorescence, 19.8-fold, 16.7-fold, and 1.9-fold higher than the PheoA-CSSE, PheoA-CSSE/NIR, and PheoA-CSSE-JSK groups, respectively. RNS can activate matrix metalloproteinase-2 (MMP-2) to facilitate collagen degradation. Accordingly, we evaluated MMP-2 expression and collagen content using immunofluorescence and Masson’s trichrome staining. The PheoA-CSSE-JSK/NIR group exhibited elevated MMP-2 levels and pronounced collagen degradation. Collagen breakdown presumably enhances oxygen infiltration and alleviates tumor hypoxia, as reflected by weakened HIF-1α immunofluorescence signals in the PheoA-CSSE-JSK/NIR cohort. This group also produced the highest intratumoral ROS-fluorescence intensity. Collectively, the enhanced PDT performance of PheoA-CSSE-JSK NPs under NIR irradiation originates from multiple NO-associated effects: intracellular GSH depletion, mitochondrial-respiratory-chain inhibition, and tumor-hypoxia alleviation (Figure 5C). Although our results demonstrate correlative evidence for this cascade, further biochemical experiments including gelatin zymography and in vitro MMP-2 activity assays will be required in future work to fully validate the proposed mechanistic sequence.

### 3.7. Mitochondrial Damage, Cellular Apoptosis, and In Vitro Antitumor Performance

**Figure 6:**
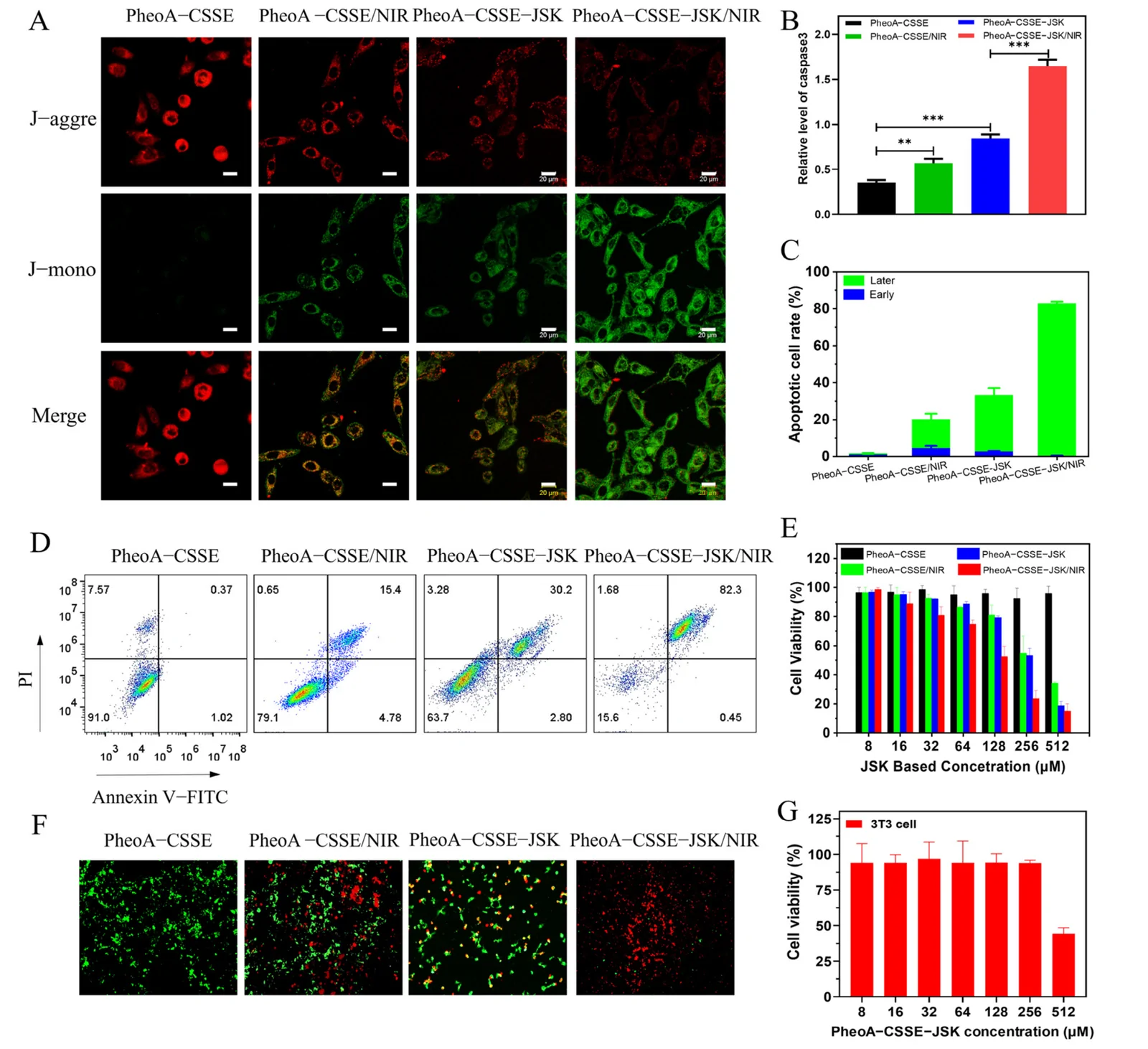
Mitochondrial injury and in vitro antitumor effects. (**A**) CLSM-acquired JC-1 fluorescence images reflecting mitochondrial-membrane-potential changes in 4T1 cells (scale bar = 20 μm), red: J-aggre, green: J-mono. (**B**) Relative cellular caspase-3 activity of 4T1 cells under different treatment conditions (n = 3). ** *p* < 0.01, and *** *p* < 0.001. (**C**,**D**) Annexin-V-FITC/PI flow-cytometry apoptosis analysis for 24 h. (**E**) 4T1 cell viability under different nanoparticle-treatment regimens at varying concentrations (48 h incubation). (**F**) Live/dead (Calcein-AM/PI) fluorescence staining of 4T1 cells (scale bar = 50 μm), red: PI, green: Calcein-AM. (**G**) Cell viability of normal 3T3 fibroblasts after nanoparticle exposure for 48 h.

A JC-1 fluorescent probe was applied to monitor mitochondrial-membrane potential. Healthy mitochondria emit red JC-1 aggregate fluorescence; upon mitochondrial-membrane depolarization, green JC-1-monomer fluorescence predominates [56]. The PheoA-CSSE-JSK/NIR group displayed the strongest green and weakest red fluorescence, indicative of severe mitochondrial-membrane damage induced by excessive ROS generation (Figure 6A). Loss of mitochondrial-membrane potential is an early hallmark of mitochondrial-dependent apoptosis [57]. Consistently, the highest cellular Caspase-3 activity was detected for PheoA-CSSE-JSK/NIR-treated 4T1 cells (Figure 6B).

Flow-cytometry-based Annexin-V-FITC/PI apoptosis quantification revealed total-apoptosis ratios of 20.18% (PheoA-CSSE/NIR) and 33.0% (PheoA-CSSE-JSK). Upon combining PheoA-CSSE-JSK with NIR irradiation, total apoptosis rose to 82.75%, dominated by late-stage apoptotic populations (82.3%, Figure 6C,D). These results demonstrate that the NO-derived biological effects significantly sensitize tumor cells toward PDT-triggered apoptosis. The CCK-8 cytotoxicity assays confirmed concentration-dependent antitumor activity in vitro. At 256 μM (JSK-based concentration), cell viabilities of PheoA-CSSE/NIR and PheoA-CSSE-JSK groups were 55.2% and 53.0%, respectively; by contrast, cell viability dropped to 23.2% for the PheoA-CSSE-JSK/NIR combination group (Figure 6E). Live/dead staining further visually verified potent tumor-cell killing within the PheoA-CSSE-JSK/NIR cohort (Figure 6F). In vitro biocompatibility toward normal 3T3 fibroblasts was satisfactory, retaining >90% cell viability even at nanoparticle concentrations up to 256 μM (Figure 6G).

### 3.8. In Vivo Biodistribution and Antitumor Therapeutic Efficacy

**Figure 7:**
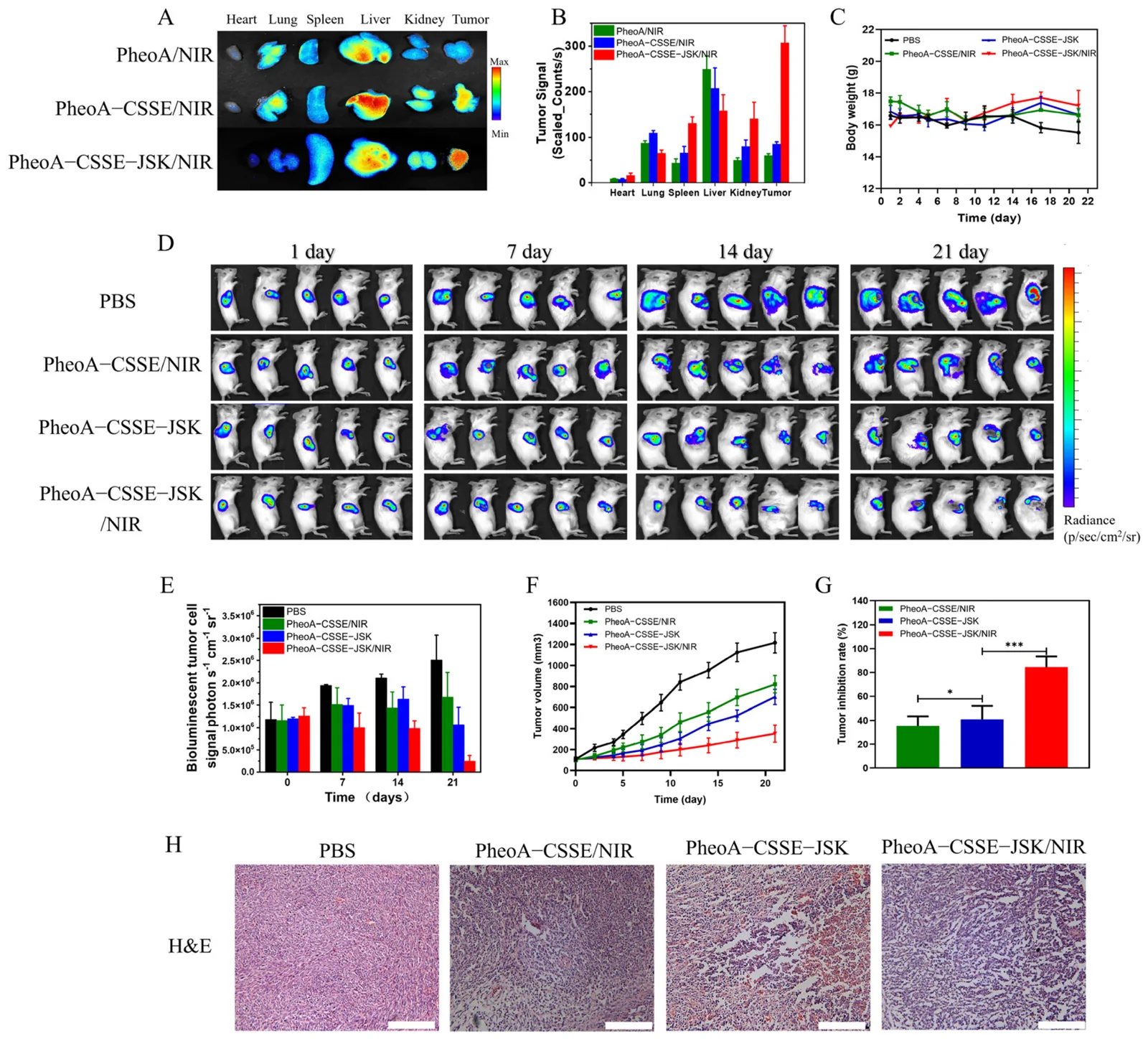
In vivo biodistribution and antitumor therapeutic performance. (**A**) Ex-vivo fluorescence images of major organs and tumors collected 24 h post-intravenous injection of free PheoA/NIR, PheoA-CSSE/NIR, or PheoA-CSSE-JSK/NIR. (**B**) Quantitative analysis of organ- and tumor-associated PheoA fluorescence signals (n = 3). (**C**) Body-weight evolution of tumor-bearing mice during treatment cycles. (**D**) Longitudinal bioluminescence imaging of 4T1-Luc-tumor-bearing BALB/c mice over 21 days (n = 5). (**E**) Quantified tumor bioluminescence intensity. (**F**) Tumor-volume growth curves. (**G**) Tumor inhibition rate of 4T1 tumor-bearing BALB/c mice after intravenous injection of PBS, PheoA-CSSE/NIR, PheoA-CSSE-JSK and PheoA-CSSE-JSK/NIR three times at 0, 4, and 7 days. (total dose: JSK = 5 mg/kg and PheoA = 7.5 mg/kg) with NIR light irradiation (660 nm, 0.2 W/cm^2^, 5 min, and 60 J/cm^2^) after each injection, * *p* < 0.05; *** *p* < 0.001. (**H**) Representative H&E-stained tumor-tissue sections from each experimental cohort (scale bar = 50 μm).

Tumor-targeted accumulation is critical for in vivo therapeutic outcomes. We tracked nanoparticle biodistribution via intrinsic PheoA autofluorescence 24 h after intravenous injection (Figure 7A,B). Owing to the EPR effect, PheoA-CSSE/NIR NPs exhibited 1.52-fold higher tumor fluorescence relative to free PheoA/NIR. Notably, the tumor-associated fluorescence signal for PheoA-CSSE-JSK/NIR NPs was 3.58-fold stronger compared with that for PheoA-CSSE/NIR. This improvement is presumably attributable to NO-driven ECM-collagen remodeling that facilitates nanoparticle tumor penetration, in accordance with the published literature. Ex vivo organ imaging showed low nanoparticle accumulation in major healthy organs, demonstrating preferential tumor-targeted biodistribution. It should be noted that although enhanced tumor accumulation was observed, our study lacks direct quantitative evidence for intratumoral nanoparticle penetration depth; cryosection-based fluorescence imaging will be pursued in future investigations to address this limitation.

This tumor-specific distribution enhances bioavailability and potential antitumor efficacy. Encouraged by the specific tumor accumulation and strong apoptotic effect of PheoA-CSSE-JSK NPs with NIR radiation in vitro, its antitumor efficiency was tested in vivo. For this, 4T1-Fluc xenografted tumor-bearing BALB/c mice with tumor volumes of up to 100 mm^3^ were used as breast cancer models. Mice were divided into four groups and received intravenous injections of the drugs every three days (four times in total), with a nanoparticle dose of 7.5 mg/kg (based on PheoA). The mice maintained stable body weight throughout the therapeutic course, implying good in vivo biocompatibility of the PheoA-CSSE-JSK nanoparticles (Figure 7C). Tumor growth was monitored via bioluminescence imaging (Figure 7D,E). The PheoA-CSSE-JSK/NIR group displayed the weakest bioluminescence signal over time, indicating pronounced tumor-growth suppression. Tumor-volume growth curves further confirmed superior therapeutic potency for this combinatorial-treatment group (Figure 7F). After 21 days of treatment, the tumor-inhibition rate of the PheoA-CSSE-JSK/NIR group reached 84.5%, substantially exceeding that of PheoA-CSSE/NIR (35.4%) and PheoA-CSSE-JSK groups (40.9%, Figure 7G). H&E staining of the tumor sections revealed extensive cellular shrinkage, nuclear fragmentation, and large necrotic regions within tumors harvested from the PheoA-CSSE-JSK/NIR-treated group (Figure 7H). Collectively, these data verify the potent synergistic in vivo antitumor therapeutic effects achieved by the PheoA-CSSE-JSK-mediated augmented PDT.

In summary, the main novelty of this work lies in the construction of an integrated multifunctional nanoplatform capable of triggering interconnected multi-modal synergistic therapeutic cascades. First, this single nano-formulation integrates GSH/GST-responsive NO release, photosensitizer delivery, intracellular GSH consumption, NO-driven collagen degradation for hypoxia alleviation, and mitochondrial-respiratory-chain inhibition. Most of the previous publications have only investigated one or two of these biological effects within separate material platforms [58,59]. Second, we systematically characterized the complete cascade of biological events: GSH-triggered prodrug activation, photosensitizer activation, intracellular GSH depletion, NO-mediated tumor-hypoxia mitigation, NO-dependent mitochondrial-respiratory-chain suppression, and their combined contribution toward PDT potentiation both in vitro and in vivo. Few of the prior reports have comprehensively evaluated this full sequential cascade within a unified nanoparticle system. Third, our modular assembly strategy provides a facile synthetic route; NO-donor and photosensitizer loading ratios can be flexibly adjusted without elaborate multi-step covalent organic synthesis. Such modular design represents practical advantages for developing multifunctional gas-combined PDT nanomedicines [60].

## 4. Conclusions

This study reports a TME-triggered dual-prodrug nanosystem PheoA-CSSE-JSK for enhanced PDT against hypoxic solid tumors. The nanosystem enables GSH-stimulated activation of self-quenched PheoA as well as sustained NO release responding to high intratumoral GSH/GST. NO-derived RNS degrades the tumor collagen matrix to alleviate hypoxia. Meanwhile, GSH depletion together with NO-mediated inhibition of the mitochondrial respiratory chain synergistically amplifies the intracellular ROS cascade. This nanotherapeutic strategy counteracts key PDT limitations including photosensitizer inactivation, hypoxia-driven therapeutic resistance, and ROS scavenging, achieving an 84.5% in vivo tumor-inhibition rate. No obvious body-weight loss was observed among treated animals. Taken together, this TME-responsive dual-prodrug nanoplatform offers a promising strategy to realize effective PDT against hypoxic solid tumors.

## Data Availability

The original contributions presented in this study are included in the article/Supplementary Materials. Further inquiries can be directed to the corresponding authors.

## Supplementary Materials

The following supporting information can be downloaded at: https://www.mdpi.com/article/10.3390/polym18202606/s1, Figure S1. ^1^H NMR of grafted block JSK-PEG (400 MHz, DMSO-d_6_), N_3_-PEG-N_3_ (400 MHz, DMSO-d_6_) and JSK (400 MHz, CDCl_3_-d_6_). The H NMR spectra were used to verify the successful conjugation of JSK onto N_3_-PEG-N_3_. Figure S2. ATR-FTIR of grafted block JSK-PEG, N_3_-PEG-N_3_ and JSK. The spectra were collected to confirm chemical structural changes after click-chemistry conjugation between JSK and N_3_-PEG-N_3_. Figure S3. ^1^H NMR of grafted block PheoA-PCL, NH_2_-PCL-N_3_ and PheoA. Spectra were acquired in CDCl_3_ at 400 MHz to verify successful conjugation of PheoA onto NH_2_-PCL-N_3_. Figure S4. ATR-FTIR of grafted block PheoA-PCL, NH_2_-PCL-N_3_ and PheoA, confirming chemical conjugation of PheoA with NH_2_-PCL-N_3_. Figure S5. ^1^H NMR of GSH responsive linker dialkynyl-Cys. The measurement (400 MHz, CDCl_3_) characterizes the chemical structure of the dialkynyl-Cys cross-linker bearing reducible disulfide bonds. Figure S6. ATR-FTIR of GSH responsive linker dialkynyl-Cys. Characteristic absorption bands for azido group (-N3-), amide groups (-CO-NH-) and disulfide bonds (-S-S-) are labeled to validate chemical structure of dialkynyl-Cys. Figure S7. Quantification of intracellular GSH/GSSG ratio after 24 h incubation of 4T1 cells with blank, PheoA-CSSE and PheoA-CSSE-JSK nanoparticles using GSH/GSSG assay kit. The assay evaluates the intracellular GSH-depleting capacity of different nano-formulations.
