# Supplementary material for: Tumor Microenvironment-Triggered Dual-Prodrug Nanoplatform for Augmented Photodynamic Therapy: Self-Quenched Photosensitizer Activation and Nitric Oxide-Mediated Sensitization
Source: Polymers (Basel). 2026 Oct 21;18(20):2606. doi: 10.3390/polym18202606 (PMC13709318; doi:10.3390/polym18202606)
Supplement: Supplementary file 1 [file polymers-18-02606-s001.zip › polymers-4631195-supplementary.pdf]

Supplementary data

Tumor Microenvironment-Triggered Dual-Prodrug Nanoplatform for Augmented Photodynamic Therapy: Self-Quenched Photosensitizer Activation and Nitric Oxide-Mediated Sensitization

Yuxuan Gu †, Jiaqi Ding †, Jialin Zhang, Xiaoxuan Liu, Zhuotao Xu, Jimin Zhang * and Xiongwei Qu *

Hebei Key Laboratory of Functional Polymers, School of Chemical Engineering and Technology, Hebei University of Technology, Tianjin 300401, China

* Correspondence: zhangjimin@hebut.edu.cn (J.Z.); xwqu@hebut.edu.cn (X.Q.)

† These authors contributed equally to this work.

Supplementary data

Characterization

The ^{1}H NMR spectra were acquired using an AVANCE 400spectrometer operating at 400 MHz. Infrared (IR) analysis was performed utilizing Fourier transform infrared spectroscopy (FTIR) with a TENSOR 27 instrument from Bruker. The crystalline structure of the samples was examined via X-ray diffractometry (XRD) using a D8 Discover system, also from Bruker. The morphology of the nanoparticles was investigated with a Talos F200S transmission electron microscope (TEM). Differential scanning calorimetry (DSC) w as conducted on a Q20 instrument, heating the samples from 20 to 150 °C in a dynamic nitrogen atmosphere at a rate of 10 °C/min. UV-vis absorption spectra were recorded using a Cary 300 Scan spectrophotometer from Varian. Fluorescence measurements were analyzed with a Leica TCS NT1 confocal laser scanning microscope (Germany) and quantitatively determined using a BD Accuri C6 flow cytometer (BD Biosciences, San Jose, CA). Histology and immunohistochemistry slides were examined with a Zeiss Axio Imager Z1 fluorescence microscope (Oberkochen, Germany).

Supplementary data

Supporting Figures

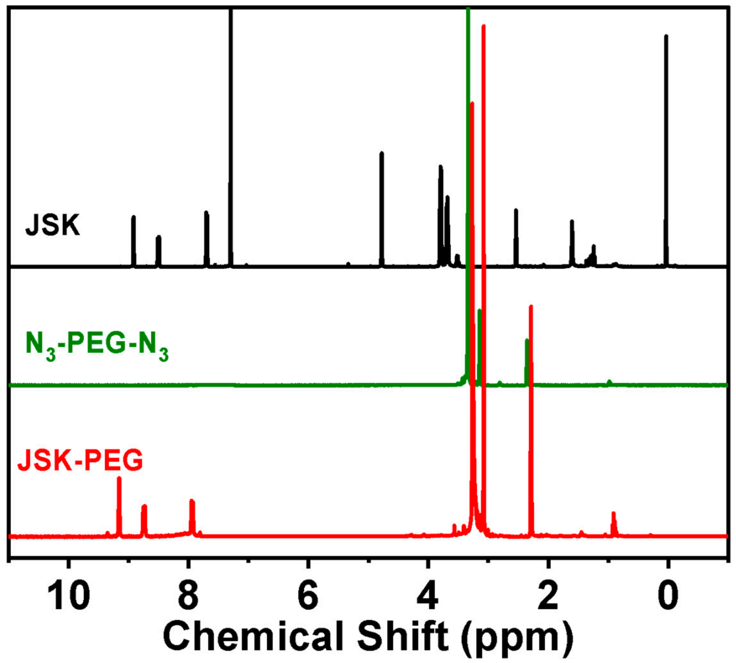

Figure S1. 1H NMR of grafted block JSK-PEG (400 MHz, DMSO-d6), N3-PEG-N3 (400 MHz, DMSO-d6) and JSK (400 MHz, CDCl3-d6). The H NMR spectra were used to verify the successful conjugation of JSK onto N3-PEG-N3.

Supplementary data

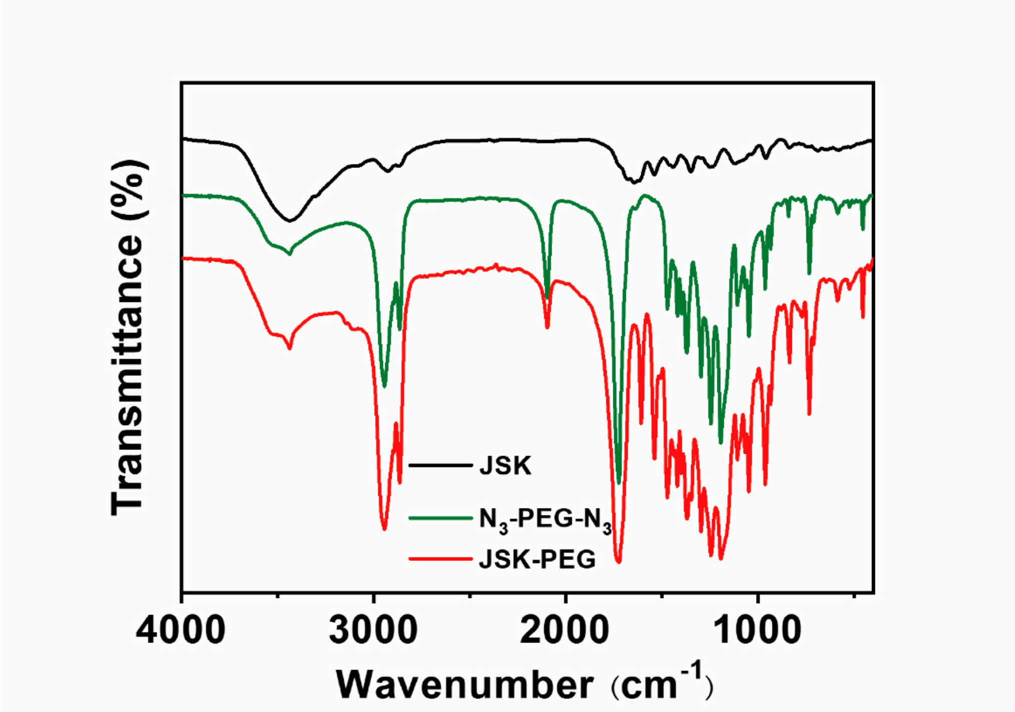

Figure S2. ATR-FTIR of grafted block JSK-PEG, N3-PEG-N3 and JSK. The spectra were collected to confirm chemical structural changes after click-chemistry conjugation between JSK and N3-PEG-N3.

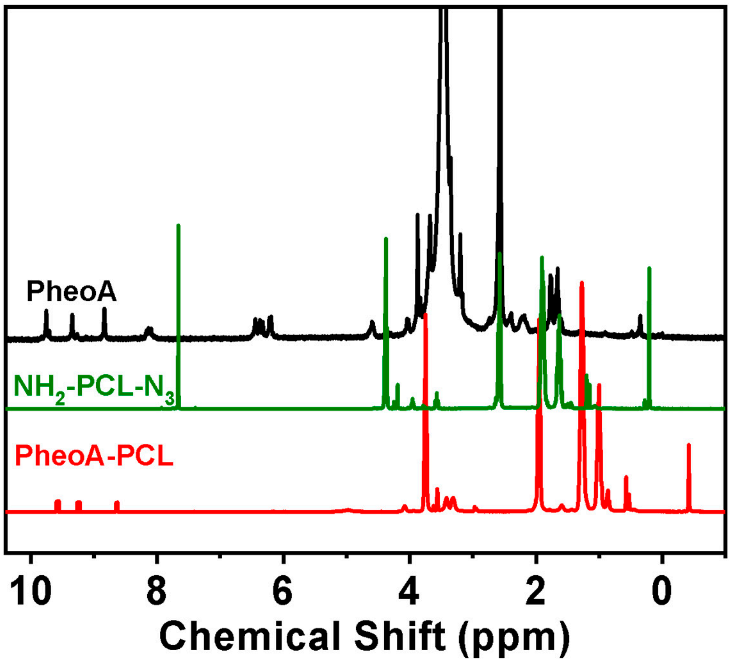

Supplementary data

Figure S3. 1H NMR of grafted block PheoA-PCL, NH2-PCL-N3 and PheoA. Spectra were acquired in CDCl3 at 400 MHz to verify successful conjugation of PheoA onto NH2-PCL-N3.

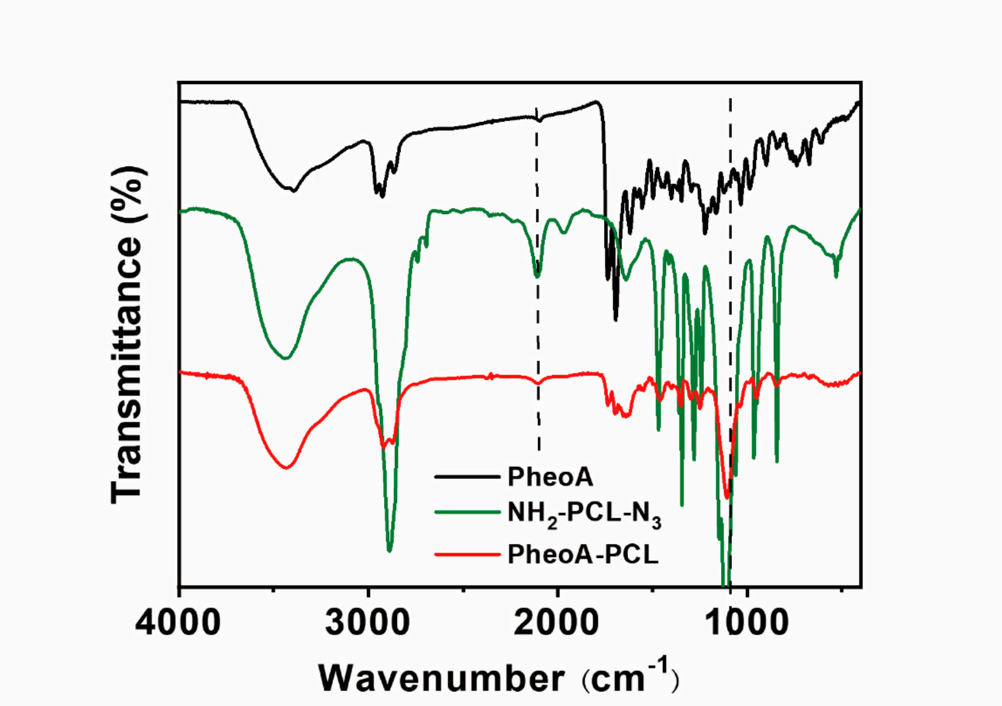

Figure S4. ATR-FTIR of grafted block PheoA-PCL, NH2-PCL-N3 and PheoA, confirming chemical conjugation of PheoA with NH2-PCL-N3.

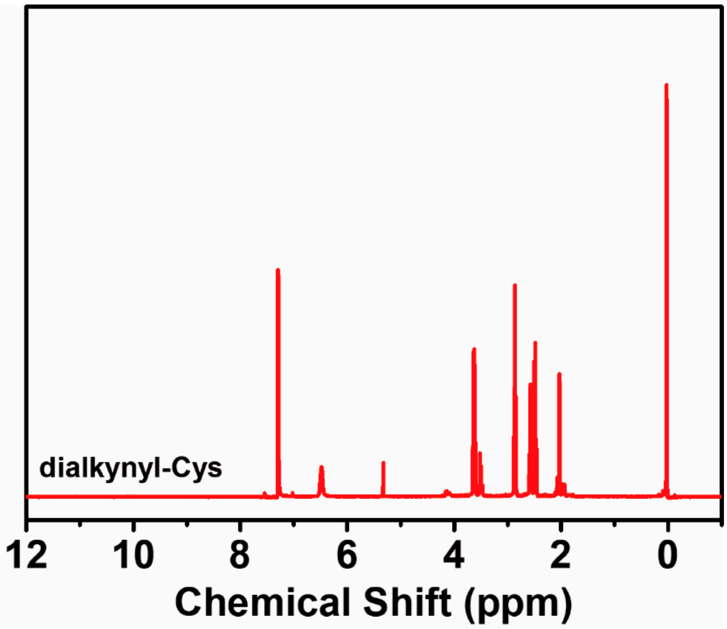

Supplementary data

Figure S5. 1H NMR of GSH responsive linker dialkynyl-Cys. The measurement (400 MHz, CDCl3) characterizes the chemical structure of the dialkynyl-Cys cross-linker bearing reducible disulfide bonds.

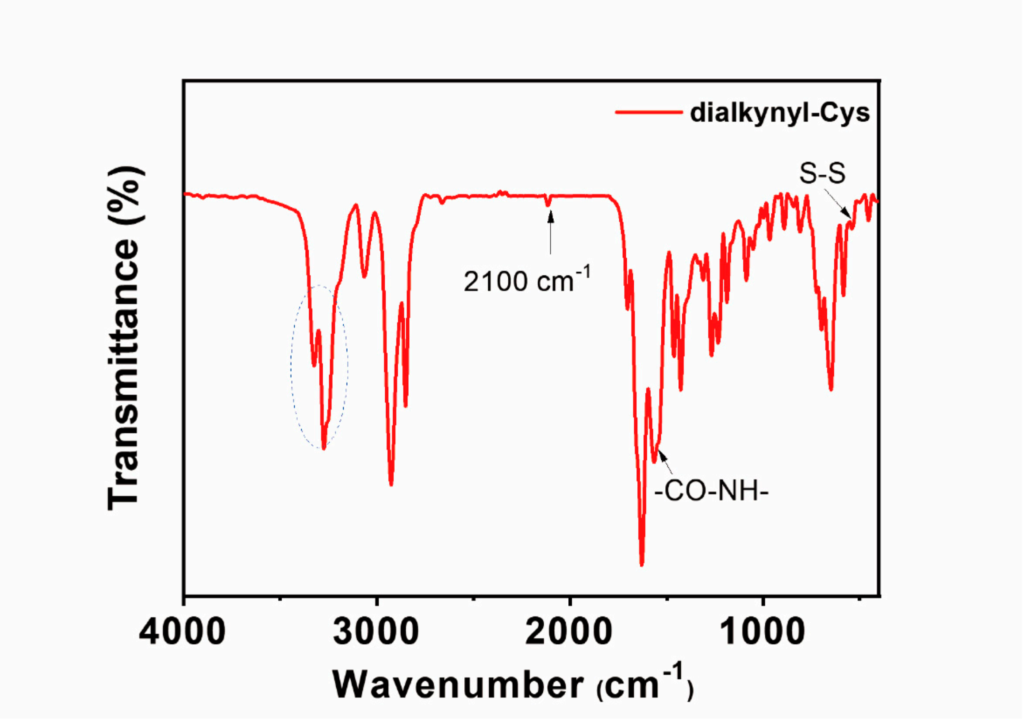

Figure S6. ATR-FTIR of GSH responsive linker dialkynyl-Cys. Characteristic absorption bands for azido group (-N3-), amide groups (-CO-NH-) and disulfide bonds (-S-S-) are labeled to validate chemical structure of dialkynyl-Cys.

Supplementary data

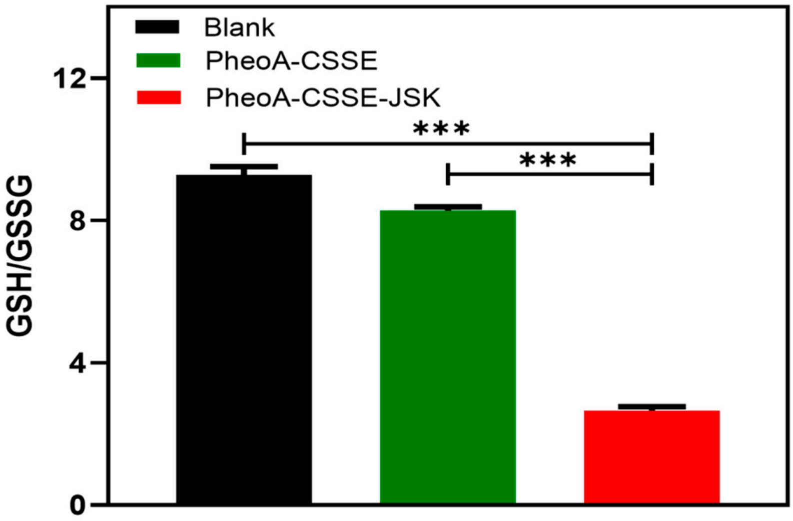

Figure S7. Quantification of intracellular GSH/GSSG ratio after 24 h incubation of 4T1 cells with blank, PheoA-CSSE and PheoA-CSSE-JSK nanoparticles using GSH/GSSG assay kit. The assay evaluates the intracellular GSH-depleting capacity of different nano-formulations.
